# Statistical control of structural networks with limited interventions to minimize cellular phenotypic diversity represented by point attractors

**DOI:** 10.1038/s41598-023-33346-1

**Published:** 2023-04-18

**Authors:** Jongwan Kim, Corbin Hopper, Kwang-Hyun Cho

**Affiliations:** Department of Bio and Brain Engineering, Korea Advanced Institute of Science and Technology (KAIST), Daejeon, 34141 Republic of Korea

**Keywords:** Control theory, Dynamic networks, Regulatory networks, Systems analysis, Cellular signalling networks, Gene regulatory networks

## Abstract

The underlying genetic networks of cells give rise to diverse behaviors known as phenotypes. Control of this cellular phenotypic diversity (CPD) may reveal key targets that govern differentiation during development or drug resistance in cancer. This work establishes an approach to control CPD that encompasses practical constraints, including model limitations, the number of simultaneous control targets, which targets are viable for control, and the granularity of control. Cellular networks are often limited to the structure of interactions, due to the practical difficulty of modeling interaction dynamics. However, these dynamics are essential to CPD. In response, our statistical control approach infers the CPD directly from the structure of a network, by considering an ensemble average function over all possible Boolean dynamics for each node in the network. These ensemble average functions are combined with an acyclic form of the network to infer the number of point attractors. Our approach is applied to several known biological models and shown to outperform existing approaches. Statistical control of CPD offers a new avenue to contend with systemic processes such as differentiation and cancer, despite practical limitations in the field.

## Introduction

Cellular phenotypes characterize cellular responses to their environment. Due to the complex dynamics underlying gene expression, cells can induce and maintain remarkably diverse phenotypes from a single genome^1–3^, referred to as cellular phenotypic diversity (CPD). CPD allows cells to react differently to a wide variety of environmental signals. Stem cells tend to have higher CPD than differentiated cells, which may be critical to forming distinct cell fates from genetically identical cells^4,5^. Conversely, CPD in cancerous cells impedes treatment. The existence of multiple phenotypes in a tumor enables certain subpopulations with drug resistant phenotypes to survive treatment^6,7^. Moreover, increased CPD in a healthy cell can be an early warning sign of cancer^5^. The pivotal role of CPD in these processes suggests that control of CPD may reveal novel therapeutics. For instance, control that reduces CPD of cancer cells may undermine drug resistance. However, the major challenge is that CPD is highly unpredictable since it emerges from complex interactions among many cellular components.

Network control theory based on systems biology is a powerful framework to analyze CPD since it specializes in complex interactions. In network control theory, network models are utilized to untangle complex interactions by describing each cellular component as a node, and interactions between nodes as edges. Network models can be primarily categorized into two frameworks: dynamical models and structural models^8–10^. Dynamical models quantify the states of nodes over time, but quantifying the interactions between components may be prohibitively difficult in practice due to the excessive number of experiments required. In contrast, structural networks are simpler to construct, since they only consider which cellular components interact without quantifying their details. This can be confirmed by comparing the size of typical databases: the OmniPath^11^ database of structural models is much larger than the Cell Collective^12^ database of dynamic models. However, landmark structural control approaches have been criticized as overlooking dynamics that are key to control^13^. Moreover, due to their limited detail, there exist fewer control techniques using structural models than dynamic models, and few of them are applicable to CPD. In response, this study takes a pioneering step in leveraging structural network models to control, and more specially reduce, CPD. Although our approach only requires a given structural network model, dynamical properties are key to CPD. Hence, a practical control approach for CPD should infer dynamical properties from a structural network, and be evaluated by its performance on an unknown dynamical model.

A Boolean model, a type of dynamical model, is assumed to accurately depict the underlying cellular dynamics. We focus on Boolean models since they are known to successfully describe numerous biological phenomena, while making minimal underlying assumptions^14–16^. In a Boolean model, each node state is either 1 or 0, where 1 indicates an activated state and 0 indicates an inactivated state. Over time, a node state changes due to the influence of its regulators, whose relationship is represented by a Boolean function. The network state, defined by the vector of node states, eventually flows into specific set of states called an attractor, within which the network will visit all states without leaving. Attractors are important in that one or more attractors uniquely correspond to each phenotype of a cell^8,17^. Since the phenotypes form CPD, the number of attractors tends to be proportional to the number of phenotypes and can be utilized to estimate CPD. A Boolean model has two types of attractors: a point attractor includes only a single network state, whereas a complex attractor includes at least two network states. In this study, the number of point attractors (NoPA) is of special interest, firstly because many phenotypes related to drug treatment are known to correspond directly to point attractors^18–22^, and secondly because point attractors are not dependent on the update scheme, whereas complex attractors vary depending on the degree of synchrony in node updates^23^. While complex attractors may be of interest for later studies, their sensitivity to the modeling framework suggests that point attractors are a better preliminary step into CPD control. As inferring NoPA directly from a dynamical network model is impractical due to the difficulties of dynamic model construction, this study instead infers NoPA from a given structural network model.

In our study, control involves selecting a set of cellular components, represented as nodes in the network, as control targets and changing their states. Similar to model framework choice, control targets should be limited by practical considerations so that the approach is feasible in application. This study considers the following three constraints. First, the number of control targets used to minimize CPD is limited since multiple control targets can compound off-target effects and increase deleterious side-effects for the patient. Second, for the convenience of manipulation, control should constantly fix the state of a control target to either 1 or 0. In other words, instead of specifying time-varying control or exact degrees of control, only control where control targets are permanently knocked-out (KO) or constitutively over-expressed (OE) should be considered. Third, which targets can be controlled may be restricted. Often there are simply no drugs that target a specific cellular component, or making one is infeasible. Overall, a control approach for CPD that accounts for these practical constraints will be widely applicable.

Among the limited number of control approaches for structural models, no studies consider all three practical constraints described above. This tendency can be observed in the two most well-known structural control studies. The first study utilizes a subset of nodes referred to as a feedback vertex set (FVS) to control all states to a desired attractor^24^. An immediate consequence of a FVS is that if the nodes of FVS are removed from the network, the network loses all cycles. The study suggests that by controlling the state of a FVS, the network model is driven to be in the corresponding attractor. One result is that fixing the states of the FVS nodes limits the model to a single global attractor, which is ideal for reducing CPD. However, the approach can violate the first and third constraints mentioned above. The first constraint is violated when the size of FVS exceeds the limit of the number of control targets. The third constraint is violated when every possible FVS happens to contain certain nodes that cannot be selected as a control target. Subsequent approaches rank the nodes within the FVS to return a smaller subset, but in the context of driving to or from a specified attractor, rather than controlling CPD^25,26^.

The second study of control on structural models is by Liu et al.^27^. They argue that controlling certain nodes identified via a maximum matching algorithm from the network structure alone can drive the model state from any initial state to a desired state, which we refer to as the maximum matching approach. As the original approach was only used to control between two states, it remains unclear if the approach can be extended to reduce CPD. However, subsequent studies have repeatedly shown these maximum matched nodes play a pivotal role in the dynamics of a cell, which suggests they may also be relevant to CPD^28,29^. Notably, the maximum matching approach also falls short of satisfying all constraints mentioned above, meaning that control may be limited to a subset of the maximum matched nodes. Although these two approaches can be utilized to evaluate the efficacy of our proposed approach, both require control that may be difficult to implement in practice.

In this study, we develop a statistical control (SC) approach that reduces CPD with practical constraints. The primary challenge remains to infer NoPA from the structural network information of a cell. To overcome this challenge, we first develop an exhaustive approach that is informative, but computationally impractical. The exhaustive approach calculates the exact average NoPA over all Boolean models that could correspond to the structural network. We then develop the SC approach, whose basic idea is similar to the exhaustive approach but more computationally efficient. Rather than an exact average, this approach predicts NoPA with a value referred to as ${\mathrm{NoPA}}_{\mathrm{pred}}$. SC first constructs an acyclic form of the network, which eases NoPA calculation by exploiting the connection between positive feedback and point attractors^30,31^. Specifically, each source state of the acyclic form that matches the sink state is sufficient to sustain a positive feedback across the whole network, which implies a distinct point attractor. A novel ensemble average value is then designed to estimate the state of each node using only structural information, by averaging over all possible functions for each node independently. For each source state in the acyclic form, the ensemble average values of the sink nodes are utilized to calculate the probability that the source state is likely to lead to a new point attractor for the unknown logic. ${\mathrm{NoPA}}_{\mathrm{pred}}$ is then given as the sum of the probabilities that each source state results in a point attractor. Finally, by comparing control candidates based on their ${\mathrm{NoPA}}_{\mathrm{pred}}$ reduction, our SC approach can infer control targets that minimize NoPA.

To evaluate the performance of the SC approach, we utilized biological networks from literature with known Boolean functions, including cortical area development, T cell differentiation, and aurora kinase A neuroblastoma networks. SC utilizes only the structure of the model, while the actual logic is utilized to calculate the true NoPA to evaluate its performance. SC is then compared to existing structural control approaches, where it consistently produces a larger reduction in the true NoPA. The proposed method successfully infers structural properties unique to NoPA reduction, further confirmed by the inability of traditional structural metrics to identify SC targets. Our approach opens the door to therapies that reduce CPD, such as subduing cancer heterogeneity to subvert drug resistance, and research to detect novel structures that reduce CPD in natural processes, such as differentiation.

## Methods

### Problem setting

To represent interactions among cellular components, a structural network model G(V, E) is given, where V is a set of nodes and E is a set of directed edges. Each edge is of the form (X, sign, Y) where sign $\in$ {‘+’, ‘−’} and X, Y $\in$ V. If the sign is ‘+’, then node X activates node Y. Conversely, if the sign is ‘−’, then node X inhibits node Y. Although it is not given, we also assume there exists a hidden true Boolean model that accurately describes the dynamics of the cell. In addition to the structure of G, this true Boolean model also specifies Boolean functions for each node to best reflect the dynamics of the cell.

The problem is to find control that minimizes NoPA of the true Boolean model (${\mathrm{NoPA}}_{\mathrm{true}})$, given several practical constraints on control. The similarity between the cell and the true Boolean model, along with the importance of point attractors described above, ensures that minimizing the ${\mathrm{NoPA}}_{\mathrm{true}}$ will reduce the CPD of the cell. The efficacy of an approach will be evaluated by comparing the similarity between the reduction in ${\mathrm{NoPA}}_{\mathrm{pred}}$ from the structural model, to that of the true Boolean model that cannot be utilized by the approach.

A control is defined as a set of nodes (control targets) and the corresponding control states that the nodes will be forced to. The following three constraints on control ensure that the approach is applicable in practice. First, the number of simultaneous control targets is limited. Second, only control methods fixing the state of control target to 0 or 1 are considered. Third, a set of nodes that cannot be controlled may be specified. These constraints ensure that the control method remains practical.

The possible candidates of the true Boolean model are trimmed by imposing certain constraints that ensure the Boolean functions are consistent with the structural model and are biologically realistic. The constraints on the Boolean functions are as follows. First, all regulators of all functions are non-spurious, meaning that each node is dependent on all of its parent nodes^32^. For example, assume that node $\mathrm{X}$ has regulators R1 and R2, the state of each node is ${s}_{\mathrm{X}}$, ${s}_{\mathrm{R}1}$, and ${s}_{\mathrm{R}2}$, respectively. If the function that specifies the state of $\mathrm{X}$, such that ${s}_{\mathrm{X}}=f\left({s}_{\mathrm{R}1}, {s}_{\mathrm{R}2}\right)$, satisfies

$$f\left( {s_{{{\text{R}}1}} , s_{{{\text{R}}2}} } \right) = \left( {s_{{{\text{R}}1}} \;and\,s_{{{\text{R}}2}} } \right)\;or\,\left( {s_{{{\text{R}}1}} \;and\;not \, \space s_{{{\text{R}}2}} } \right)$$

then ${s}_{\mathrm{X}}={s}_{\mathrm{R}1}$ regardless of the state of R2. Although R2 is said to be a regulator of X, it has no influence on ${s}_{\mathrm{X}}$. In this case, R2 is a spurious regulator for the function of X, which we assume never occurs.

Second, the Boolean functions are sign-compatible^33^. This means that if an activating (inhibiting) regulator changes from 0 to 1, the return value of the Boolean function of the node it regulates should not decrease (increase). For example, assume that node ${\text{X}}$ has regulators R$i\space \left( {i \in \left\{ {1,2, \ldots , n} \right\}} \right)$ and a function $f_{{\text{X}}}$. Then $s_{{\text{X}}}$ is determined by $s_{{\text{X}}} = f_{{\text{X}}} \left( {s_{{{\text{R}}1}} , s_{{{\text{R}}2}} , \ldots ,s_{{{\text{R}}n}} } \right)$. If regulator R1 is an activating regulator of ${\text{X}}$ (i.e. (R1,’+’, ${\text{X}}) \in {\text{E}})$, sign-compatibility ensures that any Boolean values $r_{i} \left( {i \in \left\{ {2,3, \ldots , n} \right\}} \right)$ cannot satisfy the equations

$$f_{{\text{X}}} \left( {s_{{{\text{R}}1}} = 1, s_{{{\text{R}}2}} = r_{2} , \ldots ,s_{{{\text{R}}n}} = r_{n} } \right) = 0\;{\text{and}}\;f_{{\text{X}}} \left( {s_{{{\text{R}}1}} = 0, s_{{{\text{R}}2}} = r_{2} , \ldots ,s_{{{\text{R}}n}} = r_{n} } \right) = 1.$$

Sign-compatibility ensures that the regulator R1 never acts as an inhibiting factor, meaning that the effect of R1 is consistent with the given structural model.

Lastly, each Boolean function is a nested canalizing function^34^. Each regulator of a nested canalizing function has a Boolean canalizing input value $p$ and a Boolean canalizing output value $q$. Regulators are hierarchical, where $Ri\prec Rj$ implies that $Ri$ has a higher priority than $Rj$. Higher priority regulators may determine the canalizing output value independent of the states of lower priority regulators. Specifically, if the regulator state ${s}_{Rj}$ matches its canalizing input value ${p}_{j}$, and the state of each higher priority regulator ${s}_{Ri}$ (with $Ri\prec Rj$) does not match its canalizing input value ${p}_{i}$, then the function returns the canalizing output value ${q}_{j}$ dictated by regulator $Rj$ regardless of the other regulator states. Node X has nested canalizing function ${f}_{\mathrm{X}}$, if ${f}_{\mathrm{X}}$ can be written in the following form, where the regulators $Ri \left( {i \in \left\{ {1,2, \ldots , n} \right\}} \right)$ are ordered such that $Ri \prec Rj$ if and only if $i < j$.

$$f_{{\text{X}}} \left( {s_{{{\text{R}}1}} , s_{{{\text{R}}2}} , \ldots ,s_{{{\text{R}}n}} } \right) = \left\{ \begin{gathered} q_{1} \quad if\space s_{{{\text{R}}1}} = p_{1} \hfill \\ q_{2} \quad if\space {\text{s}}_{{{\text{R}}1}} \ne p_{1} ,s_{{{\text{R}}2}} = p_{2} \hfill \\ \quad \quad \quad \quad \quad \quad \; \vdots \hfill \\ q_{n} \quad if\space s_{{{\text{R}}1}} \ne p_{1} ,s_{{{\text{R}}2}} \ne p_{2} , \ldots ,s_{{{\text{Rn}}}} = p_{n} \hfill \\ \end{gathered} \right.$$

Previous research has shown that canalizing and sign-compatible Boolean functions can accurately describe biological phenomenon^33^.

### Exhaustive control approach

We first develop an exhaustive approach to infer control targets minimizing ${\mathrm{NoPA}}_{\mathrm{true}}$. Since the exhaustive approach requires high computational complexity, the effectiveness of the method is only tested on a few examples, but the underlying idea motivates the SC approach, which is the main algorithm of this study. The basic idea of the exhaustive approach is to construct an ensemble model over all possible Boolean functions from the given structural network, and utilize its average behavior to infer control for the unknown true model.

First, an ensemble model is constructed by aggregating all possible Boolean models that conform to the given structural network model and obey the three constraints on Boolean functions in the true model (non-spurious, sign-compatible, and nested canalizing). The NoPA is calculated for each Boolean model in the ensemble model and the resulting average NoPA (${\mathrm{NoPA}}_{\mathrm{avg}}$) is calculated. We predicted that ${\mathrm{NoPA}}_{\mathrm{avg}}$ reduction and ${\mathrm{NoPA}}_{\mathrm{true}}$ reduction, for the same control target, would be correlated such that control that maximizes the ${\mathrm{NoPA}}_{\mathrm{avg}}$ reduction would tend to produce a large ${\mathrm{NoPA}}_{\mathrm{true}}$ reduction. We refer to this process as the exhaustive approach and provide more detail in Supplementary Fig. S1. This procedure incurs a high computational complexity due to the size of the ensemble model. The exhaustive approach suggested control targets which tends to produce large reductions in ${\mathrm{NoPA}}_{\mathrm{true}}$ for a few example networks. However, since its application is limited by its computational complexity, a modified method is needed for practical application to more complex network models.

### Overview of statistical control approach

The statistical control (SC) approach is developed to remedy the inefficiency of the exhaustive approach. The SC approach calculates ${\mathrm{NoPA}}_{\mathrm{pred}}$ and utilizes it to estimate ${\mathrm{NoPA}}_{\mathrm{avg}}$ from the exhaustive approach. For each control target the reduction of ${\mathrm{NoPA}}_{\mathrm{pred}}$ is calculated and utilized to compare control target candidates, since we predict that ${\mathrm{NoPA}}_{\mathrm{pred}}$ reduction and ${\mathrm{NoPA}}_{\mathrm{true}}$ reduction will be correlated. The SC approach selects the control with the largest ${\mathrm{NoPA}}_{\mathrm{pred}}$ reduction.

**Figure 1:**
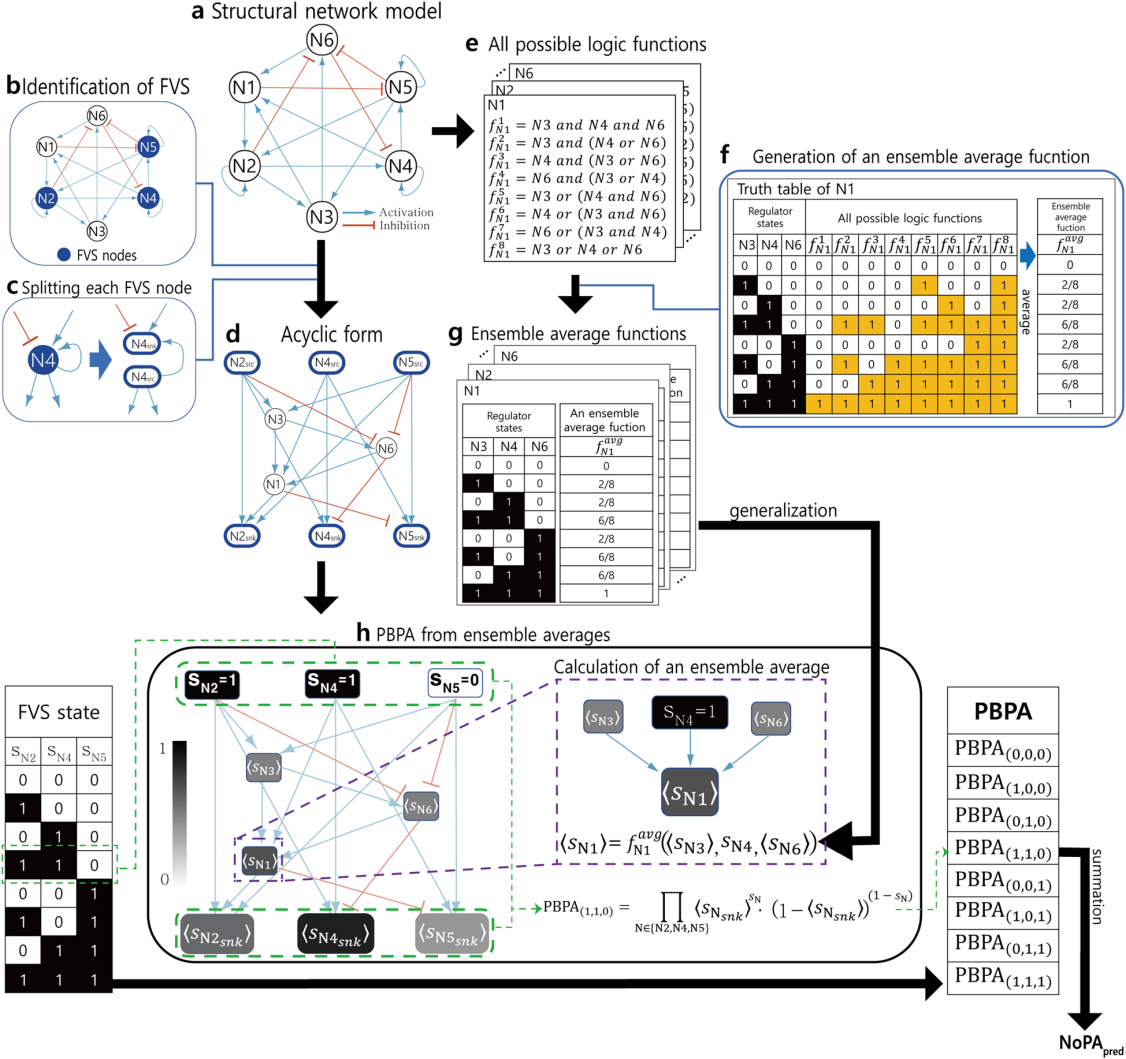
The predicted number of point attractors (${\mathrm{NoPA}}_{\mathrm{pred}}$) calculation from a structural network model. For a given structural network model, ${\mathrm{NoPA}}_{\mathrm{pred}}$ reduction is calculated. (**a**) The given structural model specifies the interactions between nodes as activation or inhibition, but not the precise logic. (**b**) First, the acyclic form of the structural network model is built. To do so, FVS nodes are identified. (**c**) Then the identified FVS nodes are split into sink (snk) and source (src) nodes. Sink nodes only retain the in-coming edges of the original FVS node, whereas source nodes only retain the out-going edges. (**d**) The acyclic form is the resulting network after splitting all FVS nodes. (**e**) Meanwhile, the ensemble average function for each node is calculated. For each node, all possible Boolean logic functions are generated, assuming non-spurious, sign-compatible, and nested canalizing functions. (**f**) For each Boolean input state, the ensemble average function (${f}^{avg}$) of a node returns the average output over all its possible Boolean logic functions. (**g**) ${f}^{avg}$ is calculated for all nodes, and then generalized to take real-valued inputs between 0 and 1, such as the ${f}^{avg}$ outputs of its regulators. (**h**) The probability of being a point attractor (PBPA) is derived by combining the acyclic form and generalized ${f}^{avg}$. For each possible set of Boolean source states, the ensemble average value ($\langle \mathrm{s}\rangle$) of each node is calculated as ${f}^{avg}$ of the states of its regulators. If a regulator is not a source node, its $\langle \mathrm{s}\rangle$ is passed as input to ${f}^{avg}$ instead of a Boolean state. PBPA is calculated as the probability that the sink node is equal to its source, where the output of the sink node is interpreted as the probability that it takes a value of 1. Finally, ${\mathrm{NoPA}}_{\mathrm{pred}}$ is calculated as the sum of PBPA over all possible source states.

The process of calculating $${\mathrm{NoPA}}_{\mathrm{pred}}$$ is summarized in Fig. 1. In this example, the structural network model is assumed to be a strongly connected component (SCC), such that there exists a directed path between every pair of nodes^35^. First, the acyclic form, which will be defined below, is constructed from the given structural network model (Fig. 1a, b, c, d). This simplifies NoPA prediction while preserving the FVS node information, which uniquely defines point attractors. Specifically, each point attractor corresponds to a network state in which the source states match the sink states of the acyclic form. Second, the ensemble average function ($${f}^{avg})$$, which will also be defined below, is calculated for each node. $${f}^{avg}$$ of a node returns the expected state, averaged over all possible Boolean functions the node can have, to infer dynamics of the unknown true model (Fig. 1e, f, g). $${f}^{avg}$$ is then applied to the nodes in the acyclic form to estimate the probability of having a point attractor when the FVS nodes of the unknown true Boolean model have specific states. The probability for a specific FVS node state to be a point attractor is referred to as the probability of being a point attractor (PBPA) (Fig. 1h). Finally, the sum of PBPA forms $${\mathrm{NoPA}}_{\mathrm{pred}}$$, which will be used to infer $${\mathrm{NoPA}}_{\mathrm{true}}$$ of the unknown true Boolean model. Each step is explained in detail below.

### Acyclic form of the network model

The acyclic form is a modified network constructed from the original structural network (Fig. 1a). The process of constructing an acyclic form is as follows. First, the FVS nodes^24^ of the network model are derived (Fig. 1b). Next, each FVS node X is separated into a corresponding source node ${\mathrm{X}}_{src}$, a node with all out-going edges of the original node, and a corresponding sink node ${\mathrm{X}}_{snk}$, a node with all in-coming edges of the original node (Fig. 1c). If the FVS node has a self-loop, then the source node and the sink node are connected by that self-loop edge. For example, if the original network contains the edge (X,’+’, X), and X is a FVS node, then the edge (${\mathrm{X}}_{src}$,’+’, ${\mathrm{X}}_{snk}$) exists in the acyclic form. The final acyclic form ends up having no cycle, due to a property of the FVS (Fig. 1d)^24^.

This structure retains the influence a FVS node has on itself, represented in the paths between the sink and source nodes corresponding to the same FVS node. Notably, in each point attractor of a model the set of FVS node states are unique^24^. Hence, the acyclic form eases point attractor inference: if the states of the source nodes match those of the corresponding sink nodes, i.e. ${s}_{{\mathrm{X}}_{snk}}={s}_{{\mathrm{X}}_{src}}$ for all nodes X in FVS, then that state is a point attractor in the original Boolean model. Moreover, since a Boolean model with acyclic form is free from feedback influence, the calculations become far simpler compared to those on the original model. The state of each node can be inferred once, without the risk of subsequent change due to feedback. Hence the acyclic form preserves the relations that are crucial for point attractors, while simplifying their prediction.

### Ensemble average function and ensemble average value

We define a novel ensemble average function ${f}^{avg}$ and ensemble average value $\langle s\rangle$ for each node to approximate the average of a node state in the ensemble model from the exhaustive approach. When regulators states of a node are given, the state of the node depends on the Boolean function, which can be different for each model in ensemble model. $\langle s\rangle$ of a node is the approximate state of the node, averaged over all possible models in the ensemble model, given a specific state for each regulator. $\langle s\rangle$ can be understood as a generalization of a state: it is possible to calculate $\langle s\rangle$ of a node when the regulator states are expressed as $\langle s\rangle$ instead of states. A novel function called the ensemble average function, symbolized as ${f}^{avg}$, is derived from all possible Boolean functions of a node to calculate $\langle s\rangle$ of that node. ${f}^{avg}$ of a node receives the states or $\langle s\rangle$ of its regulators and returns $\langle s\rangle$.${f}^{avg}$ is built through the following process. First, for each node, all possible Boolean functions are generated, which obey the three constraints about Boolean functions in the true model (non-spurious, sign-compatible, and nested canalizing). Continuing with the example network from Fig. 1a, node N1 has 8 possible Boolean functions that satisfy the constraints described as $f_{{{\text{N}}1}}^{i} \left( {i \in \left\{ {1,2, \ldots 8} \right\}} \right)$ (Fig. 1e). Other nodes of the example network also have a list of possible Boolean functions. The value of ${f}^{avg}$ for each set of regulator states is the average function value over all possible Boolean functions. The following equations express ${f}^{avg}$ when the inputs of the function are comprised of Boolean states. Let ${s}_{\mathrm{N}i}\in \{\mathrm{0,1}\} (i\in \left\{\mathrm{1,2},\dots ,m\right\})$ be the regulator states of the node. If the node has a list of possible Boolean functions $\{{f}^{1},{f}^{2},{f}^{3},\dots ,{f}^{n}\}$, then,

1 $${f}^{avg}\left({s}_{\mathrm{N}1},{s}_{\mathrm{N}2},\dots ,{s}_{\mathrm{N}m}\right)=\frac{1}{n}\sum_{i=1}^{n}{f}^{i}({s}_{\mathrm{N}1},{s}_{\mathrm{N}2},\dots ,{s}_{\mathrm{N}m})$$

For the case of node N1 in the example network, ${f}^{avg}$ of N1 has symbol ${f}_{\mathrm{N}1}^{avg}$ and is described on the right of Fig. 1f. The return value of ${f}^{avg}$ becomes $\langle s\rangle ,$ which has a real value between 0 and 1.

The domain of ${f}^{avg}$ can be generalized to encompass $\langle s\rangle ,$ which is a generalization of a Boolean node state to a real value between 0 and 1. The generalization is explained here and further formalized by Supplementary Algorithm S1. Let $0\le \langle {\mathrm{s}}_{\mathrm{Ni}}\rangle \le 1$ be the ensemble average value of the regulators $\mathrm{N}i \space(i\in \left\{\mathrm{1,2},\dots ,m\right\})$. For Boolean states $\mathrm{r}=\left({r}_{1},{r}_{2},\dots ,{r}_{m}\right) ({r}_{i}\in \left\{\mathrm{0,1}\right\}, i\in \left\{\mathrm{1,2},\dots ,m\right\})$, the ratio $\mathrm{p}$ of the Boolean states becomes

2 $$\mathrm{p}\left(\langle {\mathrm{s}}_{\mathrm{N}1}\rangle ,\langle {\mathrm{s}}_{\mathrm{N}2}\rangle ,\dots ,\langle {\mathrm{s}}_{\mathrm{Nm}}\rangle ,\mathrm{r}\right)=\prod_{i=1}^{m}{\langle {\mathrm{s}}_{\mathrm{N}i}\rangle }^{{r}_{i}}\cdot {(1-\langle {\mathrm{s}}_{\mathrm{N}i}\rangle )}^{(1-{r}_{i})}$$

This means that when ${r}_{i}=1$, multiply by the fraction that regulator $i$ is active $\langle {\mathrm{s}}_{\mathrm{N}i}\rangle$, whereas when ${r}_{i}=0$, multiply by the fraction that regulator $i$ is inactivate instead $(1-\langle {\mathrm{s}}_{\mathrm{N}i}\rangle )$. Let R be all possible Boolean state combinations of Boolean variables r, such that $\mathrm{R}=\{\mathrm{r}=\left({r}_{1},{r}_{2},\dots ,{r}_{m}\right)|{r}_{i}\in \left\{\mathrm{0,1}\right\}, i\in \left\{\mathrm{1,2},\dots ,m\right\}\}$. Then Eq. (2) can be used to generalized Eq. (1):

3 $${f}^{avg}\left(\langle {\mathrm{s}}_{\mathrm{N}1}\rangle ,\langle {\mathrm{s}}_{\mathrm{N}2}\rangle ,\dots ,\langle {\mathrm{s}}_{\mathrm{Nm}}\rangle \right)=\sum_{\mathrm{r}\in R}\mathrm{p}\left(\langle {\mathrm{s}}_{\mathrm{N}1}\rangle ,\langle {\mathrm{s}}_{\mathrm{N}2}\rangle ,\dots ,\langle {\mathrm{s}}_{\mathrm{Nm}}\rangle ,\mathrm{r}\right)\cdot {f}^{avg}(\mathrm{r})$$

Note that if each $\langle {\mathrm{s}}_{\mathrm{Ni}}\rangle$ is 0 or 1, then only one $\mathrm{p}\left(\langle {\mathrm{s}}_{\mathrm{N}1}\rangle ,\langle {\mathrm{s}}_{\mathrm{N}2}\rangle ,\dots ,\langle {\mathrm{s}}_{\mathrm{Nm}}\rangle ,\mathrm{r}\right)$ for $\mathrm{r}\in \mathrm{R}$ becomes 1 and all other $\mathrm{p}\left(\langle {\mathrm{s}}_{\mathrm{N}1}\rangle ,\langle {\mathrm{s}}_{\mathrm{N}2}\rangle ,\dots ,\langle {\mathrm{s}}_{\mathrm{Nm}}\rangle ,\mathrm{r}\right)$ become 0, reducing Eq. (3) back to Eq. (1).

To give a concrete example, let ${f}_{\mathrm{N}1}^{avg}$ be the ${f}^{avg}$ of N1 in the example network. Let $\langle s\rangle$ of the regulators of N1 be ($\langle {\mathrm{s}}_{\mathrm{N}3}\rangle$ = 0.4, $\langle {\mathrm{s}}_{\mathrm{N}4}\rangle$ = 0.5, $\langle {\mathrm{s}}_{\mathrm{N}6}\rangle$ = 0.7). Then the value of ${f}_{\mathrm{N}1}^{avg}(0.3, 0.4, 0.7)$ becomes

4 $${f}_{\mathrm{N}1}^{avg}\left(0.3, 0.4, 0.7\right)=\left(\begin{array}{c}\begin{array}{c}\mathrm{p}\left(0.3, 0.4, 0.7, \left(\mathrm{0,0},0\right)\right)*{f}_{\mathrm{N}1}^{avg}\left(\mathrm{0,0},0\right)+\\ \mathrm{p}\left(0.3, 0.4, 0.7, \left(\mathrm{1,0},0\right)\right)*{f}_{\mathrm{N}1}^{avg}\left(\mathrm{1,0},0\right)+\end{array}\\ \begin{array}{c}\vdots \\ \mathrm{p}\left(0.3, 0.4, 0.7, \left(\mathrm{1,1},1\right)\right)*{f}_{\mathrm{N}1}^{avg}(\mathrm{1,1},1)\end{array}\end{array}\right)$$

The values of ${f}_{\mathrm{N}1}^{avg}$ on the right side of Eq. (4) is the result of Eq. (1), and the value of $\mathrm{p}$ can be calculated using Eq. (2). As a result, ${f}_{\mathrm{N}1}^{avg}(0.3, 0.4, 0.7)$ is calculated to have the value

$${f}_{\mathrm{N}1}^{avg}\left(0.3, 0.4, 0.7\right)=\left(\begin{array}{c}\begin{array}{c}(\left(1-0.3\right)*\left(1-0.4\right)*(1-0.7))*0+\\ \left(0.3*\left(1-0.4\right)*(1-0.7)\right)*2/8+\end{array}\\ \begin{array}{c}\vdots \\ \left(0.3* 0.4 *0.7\right)*1\end{array}\end{array}\right) = 0.4605$$

The ensemble average functions are used with the acyclic form to calculate ${\mathrm{NoPA}}_{pred}$. Once the acyclic form is constructed, each node except source nodes is assigned ${f}^{avg}$ and used to calculate $\langle s\rangle$ (Fig. 1g). Then $\langle s\rangle$ are used to calculate ${\mathrm{NoPA}}_{pred}$, as explained below.

### ${\mathbf{N}\mathbf{o}\mathbf{P}\mathbf{A}}_{\mathbf{p}\mathbf{r}\mathbf{e}\mathbf{d}}$ calculation

${\mathrm{NoPA}}_{\mathrm{pred}}$ is designed to approximate ${\mathrm{NoPA}}_{\mathrm{avg}}$ in a computationally efficient manner using the acyclic form and $\langle s\rangle$. ${\mathrm{NoPA}}_{\mathrm{pred}}$ is calculated as the sum of PBPA over all FVS states. Let the PBPA defined on FVS state *S* be ${\mathrm{PBPA}}_{S}$, where *S* specifies the Boolean node state of each source node in the FVS. The process of ${\mathrm{PBPA}}_{S}$ calculation is as follows. First, the acyclic form of the given structural network model is constructed. Then ${f}^{avg}$ are built for each node except the source nodes, since the source node states are determined by the FVS state *S* instead. The FVS state is specified as $S = \left( {s_{{{\text{N}}1}} ,s_{{{\text{N}}2}} , \ldots ,s_{{{\text{N}}k}} } \right)$, where $\left\{ {{\text{N}}1,{\text{N}}2, \ldots ,{\text{N}}k} \right\}$ are FVS nodes and ${s}_{Ni}\in \left\{\mathrm{0,1}\right\} (i\in \left\{\mathrm{1,2},\dots ,k\right\})$. *S* is then assigned to the corresponding source nodes of the acyclic form, such that for a FVS node N, the state of source node ${s}_{{N}_{src}}$ is ${s}_{\mathrm{N}}$. Next, the $\langle s\rangle$ of each node in the acyclic form is calculated. First, $\langle s\rangle$ of nodes whose regulators are all source nodes are calculated using the states of source nodes and ${f}^{avg}$ of the node. Then $\langle s\rangle$ is calculated for each remaining node whose regulators have all calculated their $\langle s\rangle$ or are FVS source nodes. This is repeated until $\langle s\rangle$ of all nodes has been calculated. Finally, ${\mathrm{PBPA}}_{S}$ becomes

5 $$\mathrm{PBP}{\mathrm{A}}_{S}=\prod_{\mathrm{N}\in FVS nodes}{\langle {\mathrm{s}}_{{\mathrm{N}}_{\mathrm{snk}}}\rangle }^{{s}_{\mathrm{N}}}\cdot {(1-\langle {\mathrm{s}}_{{\mathrm{N}}_{\mathrm{snk}}}\rangle )}^{(1-{s}_{\mathrm{N}})}$$

${\mathrm{PBPA}}_{S}$ effectively estimates the probability that each FVS node ending state $\langle {\mathrm{s}}_{{\mathrm{N}}_{\mathrm{snk}}}\rangle$ matches its starting state ${s}_{N}$, after the effect of the other nodes in the network, which would imply a point attractor for that specific FVS state *S*^11^. Finally, ${\mathrm{NoPA}}_{\mathrm{pred}}$ is calculated as the sum of ${\mathrm{PBPA}}_{S}$ over all possible FVS states:

6 $${\mathrm{NoPA}}_{pred}=\sum_{S\in {\left\{\mathrm{0,1}\right\}}^{k}}{\mathrm{PBPA}}_{S}$$

where *k* is the number of FVS nodes. Hence, by summing ${\mathrm{PBPA}}_{S}$ over all *S*, ${\mathrm{NoPA}}_{\mathrm{pred}}$ estimates the average number of point attractors.

${\mathrm{PBPA}}_{S}$ calculation is visualized in Fig. 1h for the case of FVS state (${s}_{\mathrm{N}2}=1$, ${s}_{\mathrm{N}4}=1, {s}_{\mathrm{N}5}=0$) and no control on the example model. $\langle s\rangle$ is calculated in the order N3, N6, N1, and finally sink nodes $({\mathrm{N}2}_{snk}, {\mathrm{N}4}_{snk},$ and ${\mathrm{N}5}_{snk}$) resulting in values $\langle {\mathrm{s}}_{\mathrm{N}3}\rangle, \langle {\mathrm{s}}_{\mathrm{N}6}\rangle , \langle {\mathrm{s}}_{\mathrm{N}1}\rangle$, and $\langle {\mathrm{s}}_{{\mathrm{N}2}_{\mathrm{snk}}}\rangle , \langle {\mathrm{s}}_{{\mathrm{N}4}_{\mathrm{snk}}}\rangle$, $\langle {\mathrm{s}}_{{\mathrm{N}5}_{\mathrm{snk}}}\rangle$ respectively. The ${\mathrm{PBPA}}_{(\mathrm{1,1},0)}$ becomes $\left\langle {{\text{s}}_{{{\text{N}}2_{{{\text{snk}}}} }} } \right\rangle \cdot \left\langle {{\text{s}}_{{{\text{N}}4_{{{\text{snk}}}} }} } \right\rangle \cdot \left( {1 - \left\langle {{\text{s}}_{{{\text{N}}5_{{{\text{snk}}}} }} } \right\rangle } \right)$ which is the simplified form of Eq. (5) when the values of the variables ${s}_{\mathrm{N}}$ are set accordingly. This calculation is repeated over all possible FVS states (from (0,0,0) to (1,1,1)) in the example network to calculate the final ${\mathrm{NoPA}}_{\mathrm{pred}}$ using Eq. (6). The PBPA calculation process is also explained in the form of an algorithm in Supplementary Algorithm S2.

### Statistical control

The SC control approach selects control for which the reduction in ${\mathrm{NoPA}}_{\mathrm{pred}}$ is maximal, among permitted controls. The reduction in ${\mathrm{NoPA}}_{\mathrm{pred}}$ is measured by subtracting ${\mathrm{NoPA}}_{\mathrm{pred}}$ calculated with control from ${\mathrm{NoPA}}_{\mathrm{pred}}$ without control. ${\mathrm{NoPA}}_{\mathrm{pred}}$ for a specific control is calculated as in the previous section, except ${f}^{avg}$ of all nodes that are control targets is adjusted as follows. Each node in the control returns its target control value for ${f}^{avg}$, regardless of its regulators. For example, if node X is controlled by KO ($\mathrm{i}.\mathrm{e}.\space\mathrm{ X}=0$), then the ensemble average function of X (${f}_{\mathrm{X}}^{avg}$) is changed such that the function always returns 0 (${f}_{\mathrm{X}}^{avg}\left(r\right)=0\, \space for\, \space any\, \space r)$. As a result, $\langle{s}_{\mathrm{X}}\rangle$ will always match its control value.

**Figure 2:**
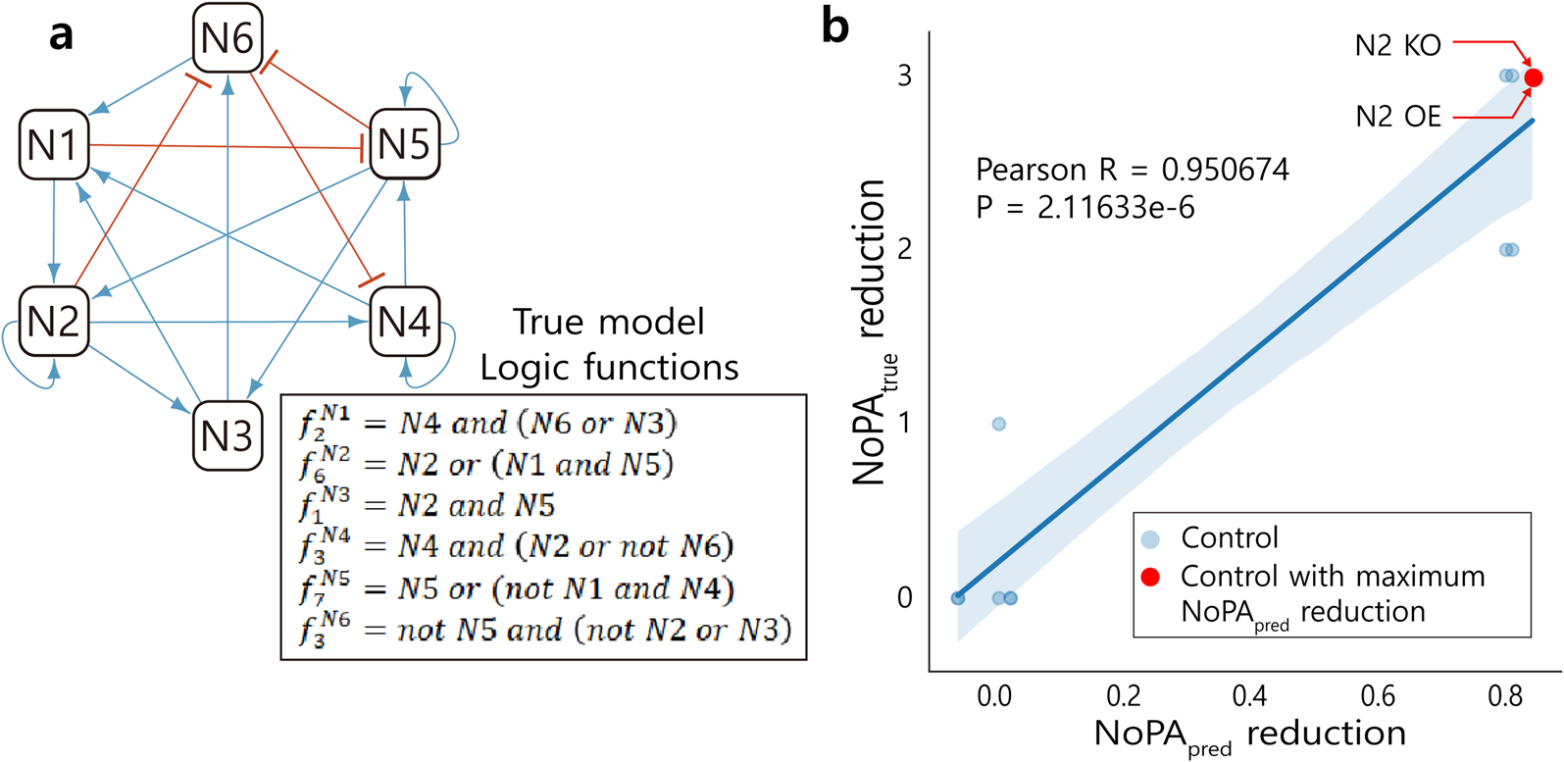
Statistical control (SC) using ${\mathrm{NoPA}}_{\mathrm{pred}}$. (**a**) The example network with the Boolean functions corresponding to the true model. The goal of SC is to reduce NoPA of the unknown true Boolean model (${\mathrm{NoPA}}_{\mathrm{true}}$) using only the structure of the network. (**b**) SC searches for controls that maximize ${\mathrm{NoPA}}_{\mathrm{true}}$ reduction on the example network. NoPA reduction is measured for each candidate control by subtracting ${\mathrm{NoPA}}_{\mathrm{pred}}$ after control from ${\mathrm{NoPA}}_{\mathrm{pred}}$ before control, where ${\mathrm{NoPA}}_{\mathrm{pred}}$ is described in Fig. 1. SC then picks the control with the largest ${\mathrm{NoPA}}_{\mathrm{pred}}$ reduction, shown in red (N2 OE and N2 KO). The ${\mathrm{NoPA}}_{\mathrm{pred}}$ reduction is then compared with that of ${\mathrm{NoPA}}_{\mathrm{true}}$, to validate their correlation.

${\mathrm{NoPA}}_{\mathrm{pred}}$ reduction should be positively correlated to ${\mathrm{NoPA}}_{\mathrm{true}}$ reduction. Figure 2a gives an example true model logic for the example network in Fig. 1. ${\mathrm{NoPA}}_{\mathrm{pred}}$ reduction for each single-node control of the example network model is calculated, along with ${\mathrm{NoPA}}_{\mathrm{true}}$ reduction of the true model in Fig. 2a. The relation is shown in Fig. 2b. The SC approach selects the control with the maximum ${\mathrm{NoPA}}_{\mathrm{pred}}$ reduction (shown in red) to infer control that minimizes CPD of a cell.

### Computational complexity

The computational complexity of SC is determined by the maximum in-degree of nodes (${\mathrm{d}}_{\mathrm{max}})$, the number of nodes (n), and the number of FVS nodes (${\mathrm{n}}_{\mathrm{FVS}})$. The computational complexity of SC is $\mathcal{O}({\mathrm{nd}}_{\mathrm{max}}!{2}^{{\mathrm{d}}_{\mathrm{max}}}+{\mathrm{nd}}_{\mathrm{max}}{2}^{{\mathrm{n}}_{\mathrm{FVS}}}$). In the process of ensemble average function calculation, the most computationally expensive step is to calculate and average all possible Boolean logic functions of a node (Eq. 1). When the in-degree of a node is $\mathrm{d}$, the number of all possible Boolean functions which obey the three constraints is at most $\mathrm{d}!{2}^{\mathrm{d}}$. Since each Boolean function, which is a nested canalizing function, can have $\mathrm{d}!$ priority permutations among regulator nodes and ${2}^{\mathrm{d}}$ canalizing input values, the worst-case combination of priorities and canalizing inputs becomes ${\mathrm{d}}_{\mathrm{max}} !{2}^{{\mathrm{d}}_{\mathrm{max}}}$. Note that in practice redundancy between priority permutations and canalizing inputs tend to lead to fewer functions. All possible logic functions are calculated and later averaged over each node, leading to another factor of $\mathrm{n}$ and a computational complexity of $\mathcal{O}({\mathrm{nd}}_{\mathrm{max}}!{2}^{{\mathrm{d}}_{\mathrm{max}}})$ for the ensemble average function calculation. To build the acyclic form, all FVS sets are identified. Since exhaustively calculating FVS is exponential in the number of nodes^36^, an approximation method is used instead^37^, which has negligible complexity relative to the rest of SC calculation. Although the approximation may not yield the minimal FVS set, the increase in complexity due to larger FVS (${2}^{{\mathrm{n}}_{\mathrm{FVS}}}$) sets is less than the cost of exhaustively calculating FVS (roughly ${2}^{\mathrm{n}}$). To calculate ${\mathrm{NoPA}}_{\mathrm{pred}}$ from the acyclic form, ${\mathrm{PBPA}}_{\mathrm{S}}$ is calculated for each possible FVS state S. There are ${2}^{{\mathrm{n}}_{\mathrm{FVS}}}$ states, and for each one, every node in the acyclic form is updated once by operating on the states of its regulators, leading to a factor of ${\mathrm{d}}_{\mathrm{max}}\mathrm{n}$. The complexity of ${\mathrm{NoPA}}_{\mathrm{pred}}$ becomes $\mathcal{O}({\mathrm{nd}}_{\mathrm{max}}{2}^{{\mathrm{n}}_{\mathrm{FVS}}})$. Since the two processes described above are sequential, the total complexity of SC is $\mathcal{O}({\mathrm{nd}}_{\mathrm{max}}!{2}^{{\mathrm{d}}_{\mathrm{max}}}+{\mathrm{nd}}_{\mathrm{max}}{2}^{{\mathrm{n}}_{\mathrm{FVS}}}$).

### Networks with many FVS, input nodes, or many SCCs

Several details complicate the above description, although the fundamental idea remains the same. First, a network model can have more than one FVS. Among the many FVSs of the model, the minimum FVS is selected to make the acyclic form. Depending on the network structure, the calculation of the minimum FVS incurs a high computational complexity. If so, an approximate algorithm can be used to find the minimum FVS set, such as the SA-FVSP-NNS algorithm^37^. However, the network can have more than one minimum FVS (or approximate minimum FVS). In this case, ${\mathrm{NoPA}}_{\mathrm{pred}}$ is calculated for each minimum FVS (or approximate minimum FVS) and averaged.

The second complication is that a network may have input nodes, which have only out-going edges. Once the states of input nodes are determined, they are assumed to remain static. The acyclic form of a network with input nodes also includes the input nodes as source nodes. Let the input condition be the vector of states of input nodes. To calculate ${\mathrm{NoPA}}_{\mathrm{pred}}$ on the networks with input nodes, ${\mathrm{NoPA}}_{\mathrm{pred}}$ for each input condition should be calculated separately first. ${\mathrm{NoPA}}_{\mathrm{pred}}$ for each input condition is calculated in the same way as before, except that the input condition is assigned to the input nodes of the acyclic form before the process. ${\mathrm{NoPA}}_{\mathrm{pred}}$ is then summed over all possible input conditions.

Finally, if the network structure is composed of more than one SCC, an additional algorithm is needed. First, the network is decomposed into its SCCs. For the network containing SCC X and SCC Y, if there is a path from a node in SCC X to a node in SCC Y, then SCC X is said to have a higher rank than SCC Y. ${\mathrm{NoPA}}_{\mathrm{pred}}$ is calculated for each SCC of the network. $\langle{s}\rangle$ of nodes of a SCC with a higher rank act as an input condition to any downstream SCC with a lower rank. The details of calculating the ensemble average influence between SCCs is explained in the Supplementary Information. These additional processes enable the SC approach to be applied to a wide range of network structures.

## Results

In practice, a researcher would apply the SC approach to a biological network for which only the structure is known. To validate that this approach can accurately estimate ${\mathrm{NoPA}}_{\mathrm{true}}$ reduction, we apply the approach to several existing biological networks with Boolean logic^38–40^. In each case, the SC approach attempts to find controls from only the structure of the network, while the given logical functions are treated as the unknown true model and utilized to evaluate the efficacy of control.

The controls selected by the SC approach are compared against controls selected by two structure-based control techniques, FVS^24^ and the maximum matching approach^27^. Since there appears to be no pre-existing structure-based control approach that minimizes NoPA, these foundational structure-based control approaches are utilized for comparison. In both FVS and the maximum matching approach, multiple sets of nodes are found. FVS control targets nodes in the union of all minimum sized FVS sets, and fixes the state of each to 0 or 1. The intersection of FVS produced too few controls to reasonably estimate the average ${\mathrm{NoPA}}_{\mathrm{true}}$ reduction, but is included in Supplementary Fig. S2 for a comprehensive comparison. Maximum matching control is defined as the set of controls targeting nodes in the union of all maximum matched nodes and fixing each state to 0 or 1. Maximum matched nodes that appear in all maximum matching sets (intersection of all maximum matched nodes) were calculated, but only contain the input nodes in these three biological networks, which are not considered valid control targets, since they typically correspond to external cues. For equitable comparison of the average ${\mathrm{NoPA}}_{\mathrm{true}}$ reduction, SC takes a number of top scoring controls equal to the number of nodes in the method it is compared against. SC control with only the top scoring control tends to exhibit a higher average (Supplementary Fig. S2), and may be leveraged in studies where an even smaller set of control candidates is desired.

### Cortical area development

**Figure 3:**
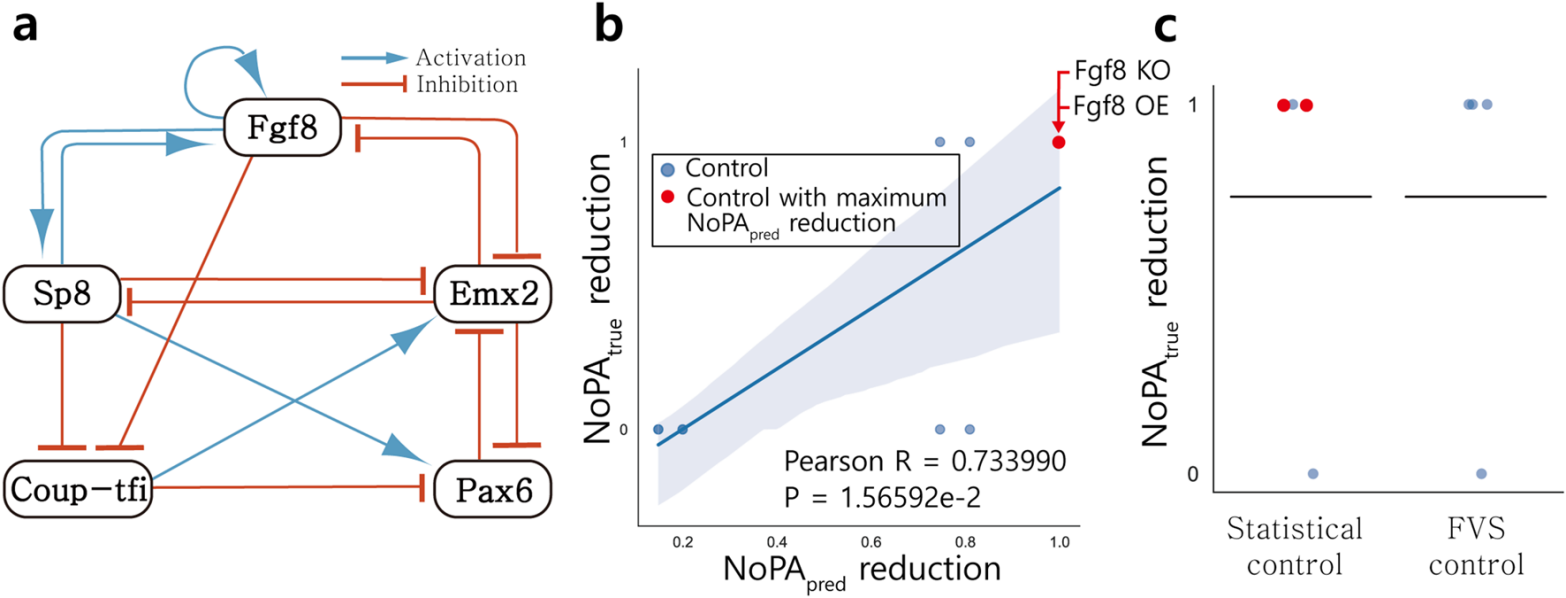
SC of cortical area development model compared to FVS and random node control. (**a**) The structural network model of cortical area development. (**b**) ${\mathrm{NoPA}}_{\mathrm{true}}$ reduction for each single-node control is calculated and compared to ${\mathrm{NoPA}}_{\mathrm{pred}}$ reduction. ${\mathrm{NoPA}}_{\mathrm{true}}$ reduction and ${\mathrm{NoPA}}_{\mathrm{pred}}$ reduction for each control are positively correlated. SC selects among the controls with the maximum ${\mathrm{NoPA}}_{\mathrm{pred}}$ reduction, shown in red (Fgf8 KO or Fgf8 OE). (**c**) The SC result for single-node control is compared to FVS control. ${\mathrm{NoPA}}_{\mathrm{true}}$ reduction of each possible control is depicted, along with a horizontal bar for the average. The top 4 SC candidates are utilized to fairly compare its average to the 4 FVS candidates. The average of ${\mathrm{NoPA}}_{\mathrm{true}}$ reduction of the two methods are the same.

The first biological model depicts cortical area development with 5 nodes and 14 edges, with no input nodes. The network structure is shown in Fig. 3a. This model analyzes the patterns of gene and protein expression in cortical development, especially patterns of the anterior–posterior axis. The 5 nodes, Fgf8, Emx2, Pax6, Coup-tfi, and Sp8 are known to make gradient patterns specifying unique coordinates for arealisation, which form specialized areas during development^38^.

${\mathrm{NoPA}}_{\mathrm{true}}$ reduction and ${\mathrm{NoPA}}_{\mathrm{pred}}$ reduction are calculated for each control that either KO (0) or OE (1) a single node. The relation between ${\mathrm{NoPA}}_{\mathrm{true}}$ reduction and ${\mathrm{NoPA}}_{\mathrm{pred}}$ reduction is plotted in Fig. 3b, which displays a positive correlation with a Pearson correlation of 0.733990 and *p* value of 1.565922e−2.

SC utilizes targets with the maximum ${\mathrm{NoPA}}_{\mathrm{pred}}$ reduction: Fgf8 KO or Fgf8 OE, both of which correspond to the maximal ${\mathrm{NoPA}}_{\mathrm{true}}$ reduction value. The top 4 SC candidates are then compared against the 4 candidates suggested by FVS control, as summarized in Fig. 3c. In this case, the two methods select the same controls. Although Fgf8 KO, Fgf8 OE, and Emx2 KO exhibit the maximal ${\mathrm{NoPA}}_{\mathrm{true}}$ reduction, Emx2 OE has a ${\mathrm{NoPA}}_{\mathrm{true}}$ reduction of 0. As a result, the expected ${\mathrm{NoPA}}_{\mathrm{true}}$ reduction of both control methods is 0.75. Since any one node is a viable maximum matched node, the maximum matching approach simply selects a node from all nodes and fixes it to 0 or 1. This naïve random node control is included in Supplementary Fig. S2, and results in an expected ${\mathrm{NoPA}}_{\mathrm{true}}$ reduction value of 0.4. In this model, reducing ${\mathrm{NoPA}}_{\mathrm{true}}$ with single-node control is most effective via either the SC or FVS approach, and less effective with maximum matched nodes.

SC isolates Fgf8 as the optimal gene to control by selecting the node with the highest ${\mathrm{NoPA}}_{\mathrm{pred}}$. Fgf8 is known as the initiating morphogen. Its activation occurs first during development, forming spatial niches that trigger the other transcription factors (the other 4 nodes of this model). Although Fgf8 is also affected by the other 4 factors, it is thought to be a particularly important factor for cortical area development^38^. SC appears to be capable of identifying key developmental nodes that reduce the number of phenotypes as cells differentiate.

### T cell differentiation

**Figure 4:**
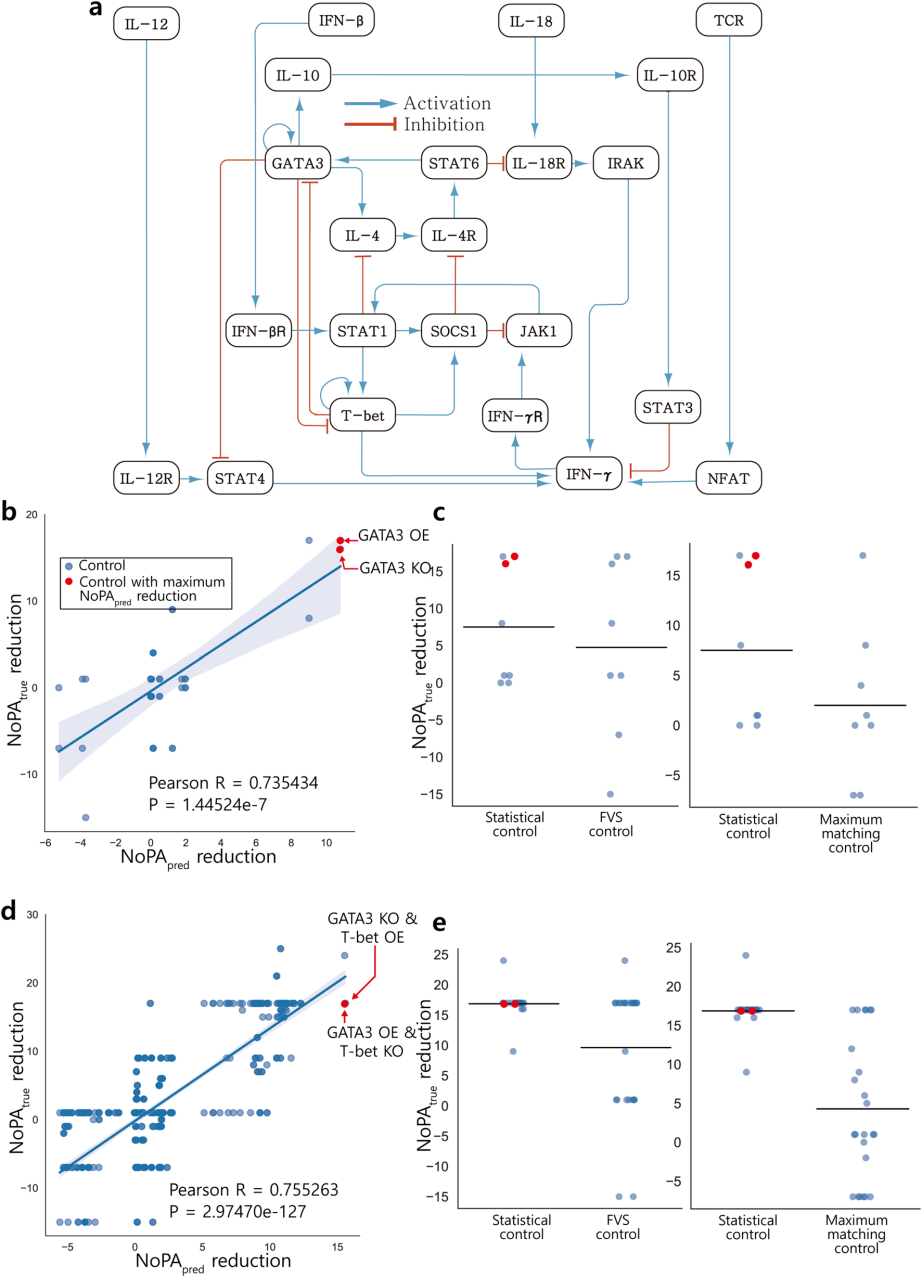
SC of T cell differentiation model compared to existing structural control methods. (**a**) The structural network model of T cell differentiation. (**b**) ${\mathrm{NoPA}}_{\mathrm{true}}$ reduction for each single-node control candidate is calculated and compared to ${\mathrm{NoPA}}_{\mathrm{pred}}$ reduction, yielding a positive correlation. SC selects among the controls with maximal ${\mathrm{NoPA}}_{\mathrm{pred}}$ reduction, shown in red (GATA3 KO or GATA3 OE). (**c**) SC is compared to several existing methods for single-node control, using the same number of SC nodes as in the comparison method. Since the network has more than one minimal FVS, FVS control represents the union of the minimal FVSs. Maximum matching control randomly selects a maximum matched node, and randomly sets it to state 0 or 1. ${\mathrm{NoPA}}_{\mathrm{true}}$ reduction of each control is depicted, along with a horizontal bar for the average. The average ${\mathrm{NoPA}}_{\mathrm{true}}$ reduction of SC is superior to all other methods. (**d**, **e**) Comparison of control methods is repeated with double-node control. SC selects GATA3 KO and T-bet OE, or GATA3 OE and T-bet KO, and is superior to other control methods after equating the number of control candidates.

The second biological network model depicts T cell differentiation and contains 23 nodes and 34 edges, including 4 input nodes. The network structure is shown on Fig. 4a. T helper cells are lymphatic cells that support the immune system and exhibit many phenotypes. This model describes the gene expression pattern of Th0, Th1, and Th2 cells which are various phenotypes of T helper cells. This model reproduces the transition from Th0 cell phenotype to Th1 cell phenotype by a large perturbation of IFN-γ. It also reproduces the transition from Th0 cell phenotype to Th2 cell phenotype by a large perturbation of IL-4^39^.

First, ${\mathrm{NoPA}}_{\mathrm{true}}$ reduction and ${\mathrm{NoPA}}_{\mathrm{pred}}$ reduction are analyzed for single-node control (Fig. 4b). The resulting Pearson correlation of 0.735434 (*p* value 1.44524e−7) indicates that the ${\mathrm{NoPA}}_{\mathrm{pred}}$ reduction is strongly correlated with ${\mathrm{NoPA}}_{\mathrm{true}}$ reduction. Next, the expected ${\mathrm{NoPA}}_{\mathrm{true}}$ reduction for each control method is calculated and compared (Fig. 4c). The top SC target is GATA3, which is also used in FVS control. FVS always contains T-bet and GATA3 due to a self-loop, as well as either JAK1 or STAT1, leading to a FVS intersection of {T-bet, GATA3} and FVS union of {T-bet, GATA3, JAK1, STAT1}. The expected ${\mathrm{NoPA}}_{\mathrm{true}}$ reduction is higher for SC than either FVS or maximum matching control, even after adjusting the number of SC controls to match the method it is compared to.

Control analysis is then repeated for double-node control (Fig. 4d). SC prioritizes GATA3 KO and T-bet OE, or GATA3 OE and T-bet KO. The expected ${\mathrm{NoPA}}_{\mathrm{true}}$ reduction for SC with double-node control is roughly double that of the other control methods (Fig. 4e). The top SC candidates GATA3 and T-bet are not key factors to transitioning the cell phenotype directly. However, previous research reveals that GATA3 activates IL-4 which triggers Th0 cells to Th2 cells^41^. This activation relation is also visible from the network structure. Another study reveals that GATA3 acts as a mediator between the IL-4 pathway and the IFN-γ pathway, which are key factors for helper T cell differentiation^42^. T-bet is an inhibiting factor of GATA3, and hence may be crucial to regulating its role in differentiation. These results suggest that SC targets reducing ${\mathrm{NoPA}}_{\mathrm{pred}}$ of the differentiation model may be fundamental to the differentiation process.

### Aurora kinase A neuroblastoma

**Figure 5:**
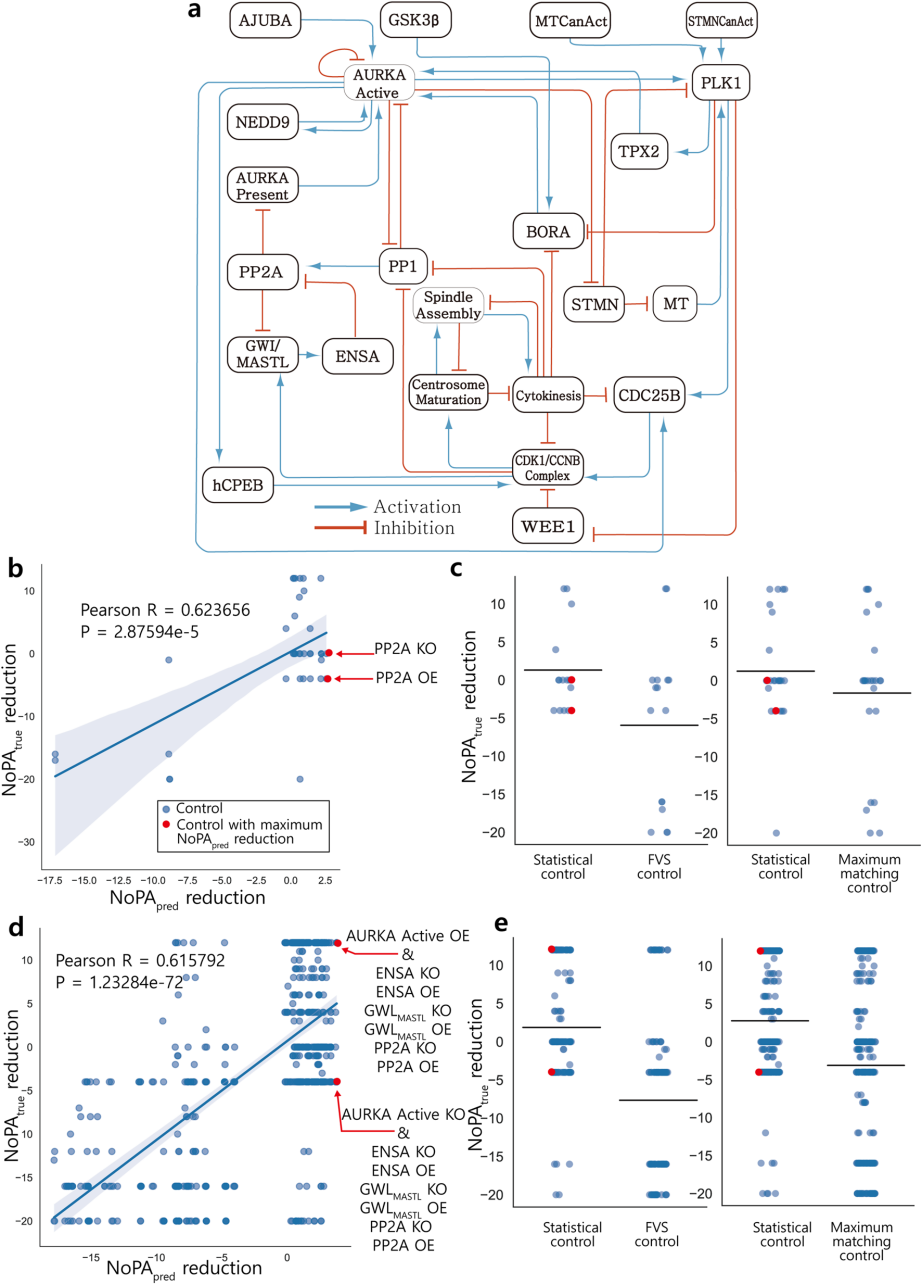
SC of aurora kinase A neuroblastoma model compared to existing control methods. (**a**) The structural network model of aurora kinase A neuroblastoma. (**b**) ${\mathrm{NoPA}}_{\mathrm{true}}$ reduction for each single-node control candidate is calculated and compared to ${\mathrm{NoPA}}_{\mathrm{pred}}$ reduction. ${\mathrm{NoPA}}_{\mathrm{true}}$ and ${\mathrm{NoPA}}_{\mathrm{pred}}$ reduction are positively correlated. SC selects the control with maximal ${\mathrm{NoPA}}_{\mathrm{pred}}$ reduction, shown in red (PP2A KO or PP2A OE). (**c**) SC is compared to several existing methods for single-node control, as described in Fig. 4. The horizontal bar representing the average resulting ${\mathrm{NoPA}}_{\mathrm{true}}$ reduction is the highest for SC. (**d**, **e**) Comparison of control methods is repeated with double-node control. SC selects randomly from all 12 controls with the highest ${\mathrm{NoPA}}_{\mathrm{pred}}$ (AURKA Active KO or OE and GWL/MASTL KO or OE, AURKA Active KO or OE and ENSA KO or OE, or AURKA Active KO or OE and PP2A KO or OE). For double-node control, SC again has higher average ${\mathrm{NoPA}}_{\mathrm{true}}$ reduction than all other approaches after equating the number of control candidates.

The Boolean network model describing aurora kinase A neuroblastoma contains 23 nodes and 43 edges, including 4 input nodes. The network structure is shown in Fig. 5a. Neuroblastoma is an extracranial solid tumor. Aurora kinase A (AURKA) is a serine/threonine kinase, whose mRNA expression is related to poor prognosis in neuroblastoma. This model analyzes the role of AURKA in neuroblastoma mitosis^40^.

${\mathrm{NoPA}}_{\mathrm{true}}$ reductions and ${\mathrm{NoPA}}_{\mathrm{pred}}$ reductions again have a positive Pearson correlation of 0.623656 (*p* value 2.87594e−5), as shown in Fig. 5b. The resulting top controls selected using the SC approach are PP2A OE or PP2A KO. The expected ${\mathrm{NoPA}}_{\mathrm{true}}$ reduction for SC is compared to those from other approaches. FVS controls consist of {AURKA Active, GWL/MASTL, CDK1/CCNB complex, spindle assembly, Centrosome Maturation, Cytokinesis, ENSA, PP2A}. In this model, the ${\mathrm{NoPA}}_{\mathrm{true}}$ reduction with SC was superior to that of both FVS and maximum matching methods (Fig. 5c).

The control methods are repeated with double-node control. ${\mathrm{NoPA}}_{\mathrm{pred}}$ reduction is again shown to be correlated with ${\mathrm{NoPA}}_{\mathrm{true}}$ reduction for double-node control (Fig. 5d). For SC, one of the two targets is always AURKA Active. The other target is either GWL/MASTL, PP2A or ENSA, and all nodes can be either fixed to 0 or 1. For double-node control, SC again has a higher expected ${\mathrm{NoPA}}_{\mathrm{true}}$ reduction than all other approaches (Fig. 5e). These results suggest that SC is more broadly applicable for finding multiple control targets or handling limitations regarding valid control targets.

The top SC single-node and double-node control candidates include four targets: AURKA Active, PP2A, ENSA, and GWL/MASTL. It is known that the AURKA Active node, which is related to AURKA gene of neuroblastoma, is related to poor prognosis^40^. Previous research reveals that PP2A induces proteasomal degradation of AURKA by dephosphorylating its Ser51 residue^43^. Both GWL/MASTL and ENSA are contained in the feedback loop involving PP2A, suggesting that this feedback is important for PP2A regulation^44,45^. SC prioritizes genes tied to poor prognosis, suggesting that treatment efficacy may be related to CPD and SC may be broadly useful for isolating important genes in cancer.

### Comparison to structural centrality

SC is compared with several metrics of centrality to test if it provides insight into dynamics that are not gleaned from traditional structural features. Positive cycles are known to correspond to point attractors, such that NoPA increases proportionately to the number of positive cycles^46^. Control could block a positive cycle by fixing a node to a specific value. Meanwhile, eigenvector centrality estimates the influence of a node on the other nodes of the network, such that nodes highly connected to other highly connected nodes have a high centrality. Unlike other centrality metrics, eigenvector centrality is applicable to directed networks with signed edges^47^. The absolute value of the eigenvector centrality is also considered, since a node rich in inhibitory interactions is still considered a strong driver of network dynamics.

**Figure 6:**
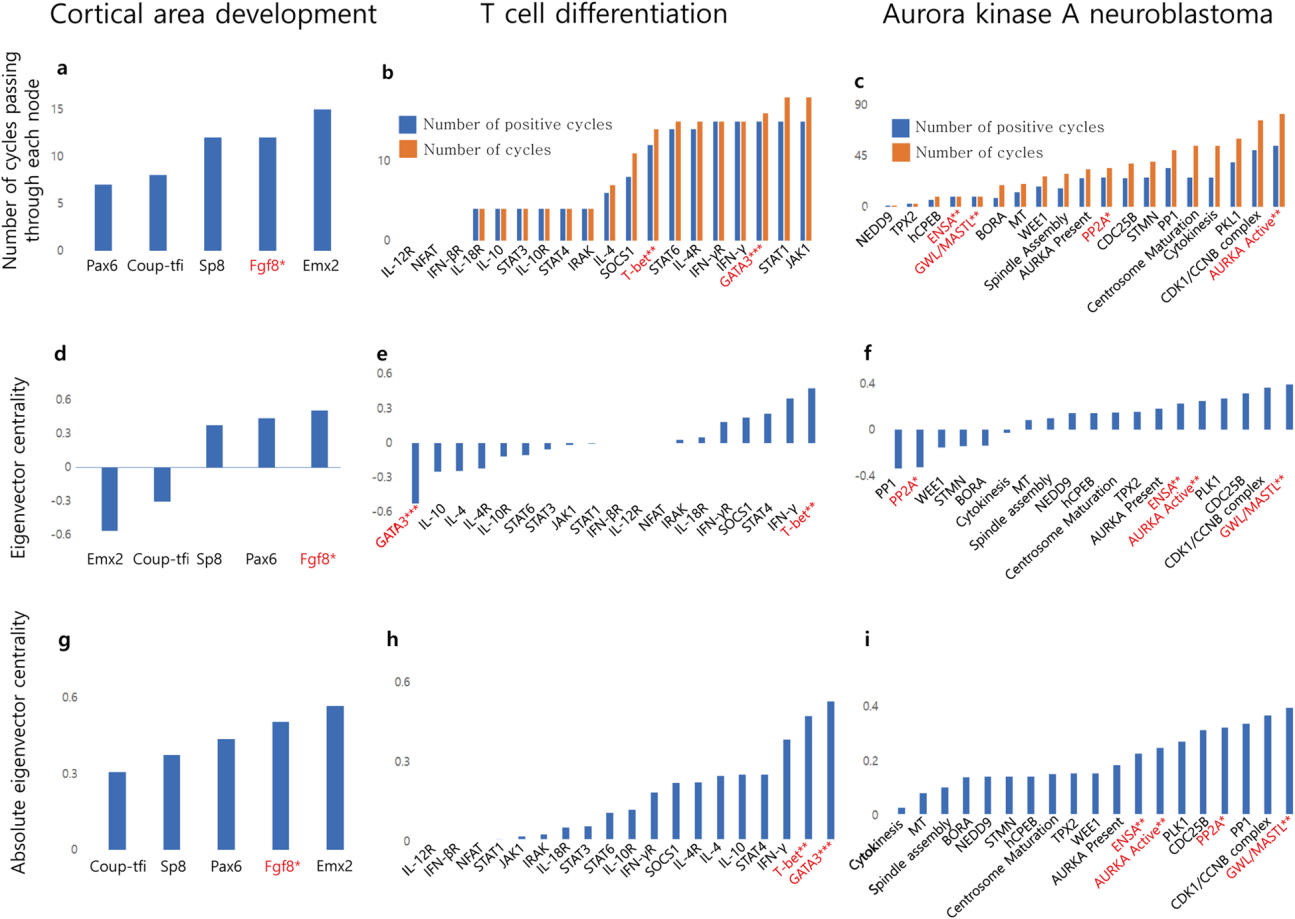
SC targets are not identifiable from structural features of centrality. For each node of each biological network several structural features are measured. Targets of statistical single-node control are marked in red with *. Targets of statistical double-node control are marked in red with **. Targets of both are in marked in red with ***. (**a**, **b**, **c**) The number of cycles and number of positive cycles passing through each node are measured. In the cortical area development model, there are only positive cycles. Targets of SCs of the three biological models tend to be involved in more cycles. However, certain notable SCs are involved in few cycles. (**d**, **e**, **f**) Although some SCs have high eigenvector centrality, others have very low eigenvector centrality. (**g**, **h**, **i**) Targets of SC tend to have high absolute eigenvector centrality. However, the nodes with maximum absolute eigenvector centrality do not always correspond to SC.

To check if SC can be inferred from positive feedback loops or node influence, the number of (positive) cycles passing through each node, its eigenvector centrality, and its absolute eigenvector centrality in all three biological models are calculated and compared (Fig. 6). The relation between (positive) cycles and control targets are analyzed (Fig. 6a, b, c). Figure 6.a shows the number of cycles of each node in cortical area development model. In this model, all feedbacks are positive cycles. Figure 6b and c show the number of cycles in the T cell differentiation model and aurora kinase A neuroblastoma model, respectively. Although SC targets tend to have a large number of cycles, the node with the most cycles is rarely a control target, and some control targets are involved in few cycles, such as GWL/MASTL and PP2A in the aurora kinase A neuroblastoma model. Hence the number of cycles or positive cycles cannot specify the control target of SC.

Eigenvector centrality metrics are also related, yet distinct from SC. While several SC targets exhibit the highest eigenvector centrality value, other targets exhibit very low eigenvector centrality values (Fig. 6d, e, f). Absolute eigenvector centrality exhibits higher correlation with targets of SC (Fig. 6g, h, i). However, it does not always specify the top SC target, implying that neither version of eigenvector centrality is sufficient to specify the SC targets. These structural centrality metrics suggest that, while SC is related to structural features, it cannot be inferred from them.

## Discussion

This study aims to develop a control technique to minimize CPD of a cell from a given structural network model. Practical restrictions may constrain the total number of control targets, which nodes are valid targets, and the granularity of control. Although the control technique is limited to the structural network, an unknown true Boolean model is assumed to describe the biological phenomenon of a cell. Although all attractors are of interest, as a preliminary step into CPD control we focus on point attractors for their known correspondence to biological phenomena and robustness with respect to the choice of modeling framework^18–23^. In this context, control that minimizes ${\mathrm{NoPA}}_{\mathrm{true}}$ is sufficient to reduce the CPD of the cell. To find the control targets from the given network structure, ${\mathrm{NoPA}}_{\mathrm{pred}}$ reductions in response to each candidate control are compared. We hypothesized that ${\mathrm{NoPA}}_{\mathrm{pred}}$ reduction would be positively correlated with ${\mathrm{NoPA}}_{\mathrm{true}}$ reduction. If so, SC can reliably reduce ${\mathrm{NoPA}}_{\mathrm{true}}$ by selecting the control with the largest ${\mathrm{NoPA}}_{\mathrm{pred}}$ reduction. To evaluate our approach, SC approach is applied to three biological models and compared with existing structural control approaches. Indeed, in all three biological examples, ${\mathrm{NoPA}}_{\mathrm{pred}}$ reduction is positively correlated with ${\mathrm{NoPA}}_{\mathrm{true}}$ reduction (Figs. 3b, 4b, d, 5b, d).

Notably, controls targeting the same set of nodes with the opposite fixed state values for each node have the same ${\text{NoPA}}_{{{\text{pred}}}}$ reduction. As a result, SC always selects both KO and OE for a given node. This is due to considering all possible Boolean logic functions for a node. If a node can have the Boolean logic f, then the node can also have a dual logic ${\text{f}}^{{\text{d}}}$ such that ${\text{f}}^{{\text{d}}} \left( {x_{1} ,x_{2} , \ldots ,x_{n} } \right) = \neg {\text{f}}\left( {\neg x_{1} ,\neg x_{2} , \ldots ,\neg x_{n} } \right)$ for all $x_{i}$ with $i \in \left\{ {1,2, \ldots ,n} \right\}$^48^. This duality can be checked in Fig. 1e. If a Boolean model X is composed of dual functions of a node of a Boolean model Y, then the model X will be referred to as a dual model of Y. For Boolean model state ${\text{A}} = \left( {a_{1} ,a_{2} , \ldots ,a_{m} } \right)$, let the inverted state of state A be $\neg {\text{A}} = \left( {1 - a_{1} ,1 - a_{2} , \ldots ,1 - a_{m} } \right)$. By the property of dual functions, if state A of model X is changed to state B, then $\neg {\text{A}}$ of the dual model Y is changed to the inverted state of B. Likewise, a point attractor of the model is an inverted state of a point attractor of its dual model. As such, KO on a node of the model has same NoPA effect to OE on the same node of the dual model. Since ${\text{NoPA}}_{{{\text{pred}}}}$ is dependent on $f^{avg}$ which is affected by the duality of the Boolean function list, KO and OE also have same effect on the ${\text{NoPA}}_{{{\text{pred}}}}$. If researchers can use prior knowledge about the dynamics of the true model, then the Boolean logic lists can be fine-tuned to reflect the partial information and break the Boolean logic duality to distinguish KO and OE^49^.

The analysis of NoPA of a model can also be interpreted using information theory. The states of the model can be viewed as information. However, the state history of a model is difficult to infer from the attractor, meaning the information outside of the attractor is mostly lost. Then, the number of attractors, or NoPA, of the model is its information capacity. The state of a model with larger information capacity may be more uncertain to an external observer, since there are more possible attractors it could occupy. The structure and logic of a network also contribute to information: Boolean functions tend to be irreversible in that the regulator states are not known from the output state. As a result, higher in-degree may contribute to information loss, whereas higher out-degree may contribute to information preservation by increasing redundancy. Hence, future work could integrate insights from information theory to improve SC, or utilize SC to provide information-theoretic insight.

SC for CPD reduction can also synergize with drug treatment for complex diseases such as cancer. Cancer cells typically exhibit high CPD, which is known to cause drug resistance: since each subpopulation reacts differently to biochemical perturbation, higher heterogeneity increases the risk of a resistant subpopulation^1^. Subpopulations that are resistant to the drug survive and proliferate. Although other drugs can be effective against this resistant phenotype, in a highly heterogeneous population, another subpopulation is likely to be resistant to this new drug. CPD reducing control can be leveraged to reduce heterogeneity of a tumor, decreasing the probability of a resistant subpopulation, and rendering it susceptible to subsequent drug treatment.

SC was utilized to find NoPA reducing targets in models for development, differentiation, and cancer. Each target was found to have an important role in the corresponding biological phenomenon. In the cortical area development model, Fgf8 is an arealisation initiating morphogen. GATA3 and T-bet are critical to IL-4 and IFN-γ dynamics in the T cell differentiation model. Targets of the aurora kinase A neuroblastoma model are key to AURKA regulation, whose activation is known to correspond to poor prognosis. These results suggest that SC of CPD is a novel approach that can be widely applied to reveal key regulators of biological phenomena.

## Supplementary Information

Supplementary Information.

## Data Availability

The datasets generated and analyzed during the current study are available from the corresponding author on reasonable request.

## Abbreviations

SC: Statistical control
CPD: Cellular phenotypic diversity
NoPA: Number of point attractors
${\text{NoPA}}_{{{\text{true}}}}$: NoPA of unknown true Boolean model
$${\text{NoPA}}_{{{\text{avg}}}}$$: Average NoPA
$${\text{NoPA}}_{{{\text{pred}}}}$$: Predicted NoPA
PBPA: Probability of being a point attractor
$f^{avg}$: Ensemble average function
$\left\langle {\text{s}} \right\rangle$: Ensemble average value
FVS: Feedback vertex set
