# Supplementary material for: Statistical control of structural networks with limited interventions to minimize cellular phenotypic diversity represented by point attractors
Source: Sci Rep. 2023 Apr 18;13:6275. doi: 10.1038/s41598-023-33346-1 (PMC10113376; doi:10.1038/s41598-023-33346-1)
Supplement: Supplementary file 1 — Supplementary Information. [file 41598_2023_33346_MOESM1_ESM.pdf]

Supplementary Information

Statistical Control of Structural Networks with Limited Interventions to Minimize Cellular Phenotypic Diversity Represented by Point Attractors

Jongwan Kim, Corbin Hopper, Kwang-Hyun Cho[1*]

[1] Department of Bio and Brain Engineering, Korea Advanced Institute of Science and Technology (KAIST), Daejeon 34141, Republic of Korea

*Author for correspondence (KHC: ckh@kaist.ac.kr)

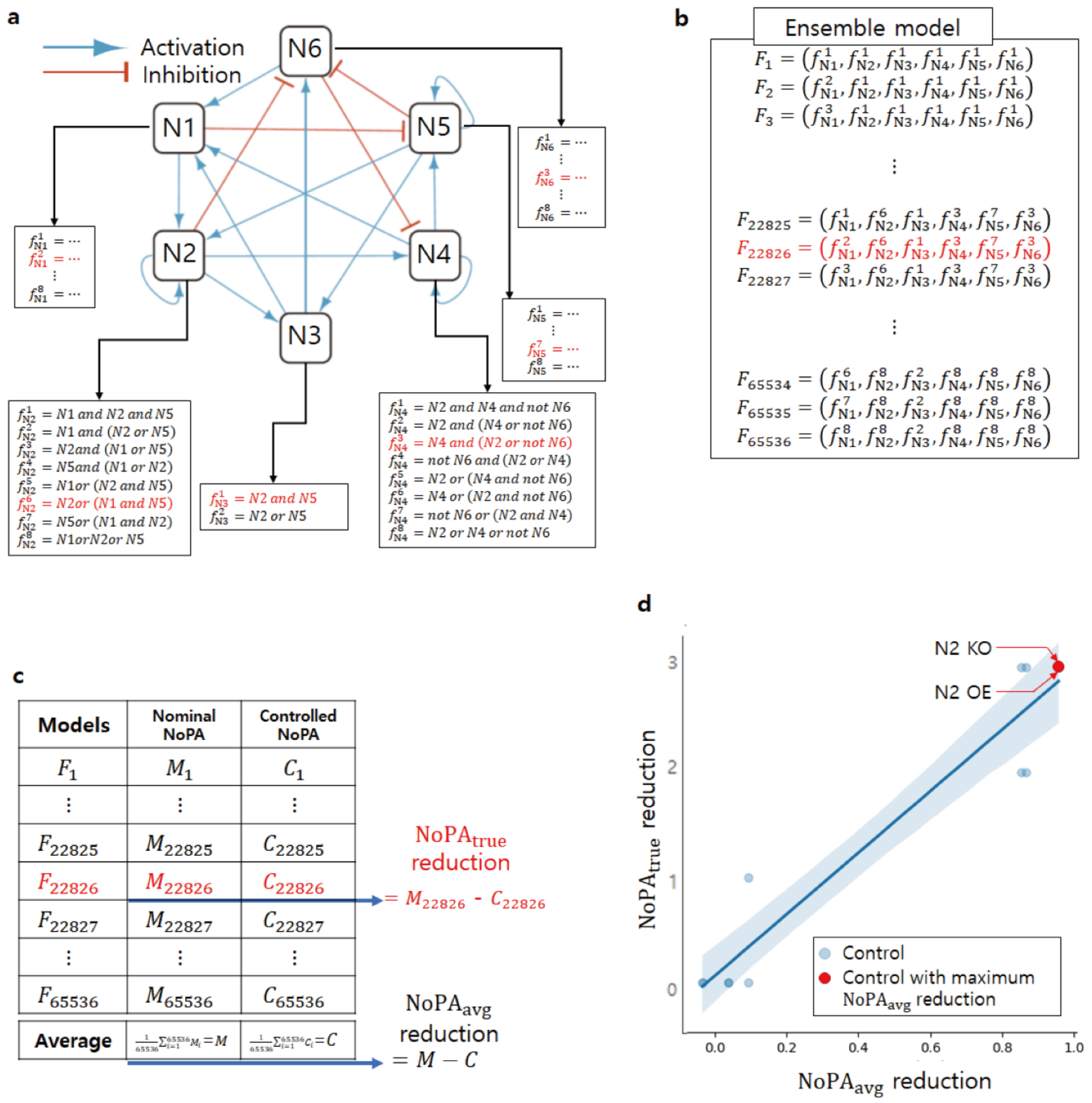

Supplementary Figure S1. Exhaustive approach

The exhaustive approach estimates the change in Cellular phenotypic diversity (CPD) due to control by averaging over an ensemble of possible Boolean functions. (**a**) Each node in the example network has a list of possible Boolean functions, given the network structure. A single dynamical model corresponds to selecting one Boolean function for each node. In this case, the functions marked in red correspond to the true model. (**b**) The ensemble model contains all possible dynamical network models that can be made. (**c**) For each dynamical model in the ensemble, calculate the number of point attractors (NoPA). The nominal NoPA is calculated without any control, whereas the controlled NoPA is calculated after

control. The NoPA reduction is obtained by subtracting the controlled NoPA from the nominal NoPA. Reductions of averaged NoPA ($\mathrm{NoPA}_{\mathrm{avg}}$) are calculated. (**d**) This $\mathrm{NoPA}_{\mathrm{avg}}$ reduction is used to select the control that induces the largest $\mathrm{NoPA}_{\mathrm{true}}$ reduction.

Supplementary Explanation. $\mathbf{NoPA_{pred}}$ calculation for network with many SCCs. First, the network is decomposed into strongly connected components (SCC). For SCC X and SCC Y of the network, if there exists a path starting from a node in SCC X and ending at a node in SCC Y, then SCC X is said to influence SCC Y. If SCC X influences SCC Y without passing through a third SCC, then certain nodes in SCC X act as input nodes to SCC Y. Once the ensemble average values ($\langle s \rangle$) of nodes in SCC X are determined, the $\mathrm{NoPA_{pred}}$ of SCC Y can be calculated in the same way as for network with input nodes. The problem is that the $\langle s \rangle$ of the nodes in SCC X is not unique. For each FVS state, the probability of being a point attractor (PBPA) of SCC X is calculated. For each such PBPA calculation, the $\langle s \rangle$ of node is calculated. As a result, a node can have as many $\langle s \rangle$ as the number of FVS states. For FVS state S, let the ensemble average value of node X ($\langle s_\mathrm{X} \rangle$) be $\langle s_\mathrm{X} \rangle_\mathrm{on\ S}$. And let the PBPA for FVS state S be $\mathrm{PBPA_S}$. To combine many $\langle s \rangle$ of a node, the weighted average is calculated.

$$\langle s_\mathrm{X} \rangle_\mathrm{overall} = \sum_{\mathrm{S} \in FVS\ states} \frac{\mathrm{PBPA_S}}{\mathrm{PBPA}} \langle s_\mathrm{X} \rangle_\mathrm{on\ S}$$

Where $\mathrm{PBPA} = \sum_{s \in FVS\ states} \mathrm{PBPA_s}$. $\langle s_\mathrm{X} \rangle_\mathrm{overall}$ is the average of $\langle s_\mathrm{X} \rangle_\mathrm{on\ S}$ weighted by $\mathrm{PBPA_S}/\mathrm{PBPA}$ and is called overall ensemble average ($\langle s \rangle_{overall}$). The $\langle s \rangle_{overall}$ of all nodes in SCC X are calculated and those with edges to SCC Y are used as input state of SCC Y.

Starting from SCCs that are not influenced by any other SCC, the $\langle s \rangle_{overall}$ of nodes are calculated. The calculated $\langle s \rangle_{overall}$ of these SCCs are used as input conditions for SCCs they influence. The $\mathrm{NoPA_{pred}}$ of each SCCs are calculated and multiplied. For instance, if a network contains SCC X and SCC Y, and $\mathrm{NoPA_{pred}}$ of SCCs are 3 and 4 respectively, the multiplied value 3·4=12 becomes the final $\mathrm{NoPA_{pred}}$ of the network.

Supplementary Algorithm S1. Generalization of ensemble average function. Ensemble average function is defined on a domain of vectors of Boolean states. it is generalized to the domain of vectors of real values between 0 and 1.

```
procedure EnsenbleAverageFunctionGeneralized(node, a)
    #[a_1, a_2, ..., a_m] == a
    ensemble_average ← 0
    m ← Length(a)
    for r in AllBooleanStates(m)
        p ← ProbabilityOfState(a, r)
        ensemble_average_of_r ← EnsembleAverageFucntion(node, r)
        ensemble_average ← ensemble_average + ensemble_average_of_r*p
    end for
    Return ensemble_average
end procedure

procedure ProbabilityOfState(a, r)
    #[a_1, a_2, ..., a_m] == a|
    #[r_1, r_2, ..., r_m] == r
    Return ∏_i^m a_i^{r_i} * (1 − a_i)^{(1−r_i)}
end procedure

procedure AllBooleanStates(m)
    states ← list()
    if m==1 then
        state_1 ← list(), state_0 ← list()
        state_1.append(1), state_0.append(0)
        states.append(state_1), states.append(state_0)
        Return states
    end if
    states_to_combine ← AllBooleanStates(m-1)
    for state in states_to_combine
        state_1 ← copy(state), state_0 ← copy(state)
        state_1.append(1), state_0.append(0)
        states.append(state_1), states.append(state_0)
    end for
    Return states
end procedure
```

Supplementary Algorithm S2. Probability of being point attractor (PBPA) calculation. For each FVS state, the PBPA is calculated. FVS_dictionary, one of the inputs of the procedure ProbabilityOfBeingPointAttractor, maps each FVS node to the states of its corresponding source node. The ensemble average of each node is calculated using the generalized ensemble average function. The ensemble averages of sink nodes are then compared to the state of corresponding source nodes.

```
#dictionary is data structure mapping key to value
#if x is dictionary and has key, value pair (y,z), x[y] == z
procedure ProbabilityOfBeingPointAttractor(nodes_of_acyclic_form, FVS_dictionary)
    #FVS_dictionary[node] == state_of_node
    ensemble_average_dictionary ← dictionary()
    for node in keys(FVS_dictionary)
        source_node ← GetSourceNode(node)
        ensemble_average_dictionary[source_node] ← FVS_dictionary[node]
    end for
    while keys(ensemble_average_dictionary) != nodes_of_acyclic_form
        node ← SelectNode(ensemble_average_dictionary, nodes_of_acyclic_form)
        regulator_nodes ← GetRegulators(node)
        ensemble_average_of_regulators ← list()
        for regulator_node in regulator_nodes
            ensemble_average_of_regulators.append(ensemble_average_dictionary[regulator_node])
        end for
        ea_node ← EnsembleAverageFucntionGeneralized(node, ensemble_average_of_regulators)
        ensemble_average_dictionary[node] ← ea_node
    end while
    Return MultiplyEAs(ensemble_averge_dictionary, FVS_dictionary)
end procedure

procedure SelectNode(node_ea_dictionary, all_nodes)
    for node in all_nodes
        if not(node ∈ keys(node_ea_dictionary))
            regulator_nodes ← GetRegulators(node)
            if regulator_nodes ⊆ keys(node_ea_dictionary)
                if regulator_nodes ⊆ keys(node_ea_dictionary)
                    Return node
                end if
            end if
        end if
    end for
end procedure

procedure MultiplyEAs(ea_dictionary, FVS_dictionary)
    ea_multiplied ← 1
    for node in keys(FVS_dictionary)
        sink_node ← GetSinkNode(node)
        ea_sink_node ← ea_dictionary[sink_node]
        if FVS_dictionary[node] == 1
            ea_multiplied ← ea_multiplied * ea_sink_node
        else if FVS_dictionary[node] == 0
            ea_multiplied ← ea_multiplied * (1-ea_sink_node)
        end if
    end for
    Return ea_multiplied
end procedure
```

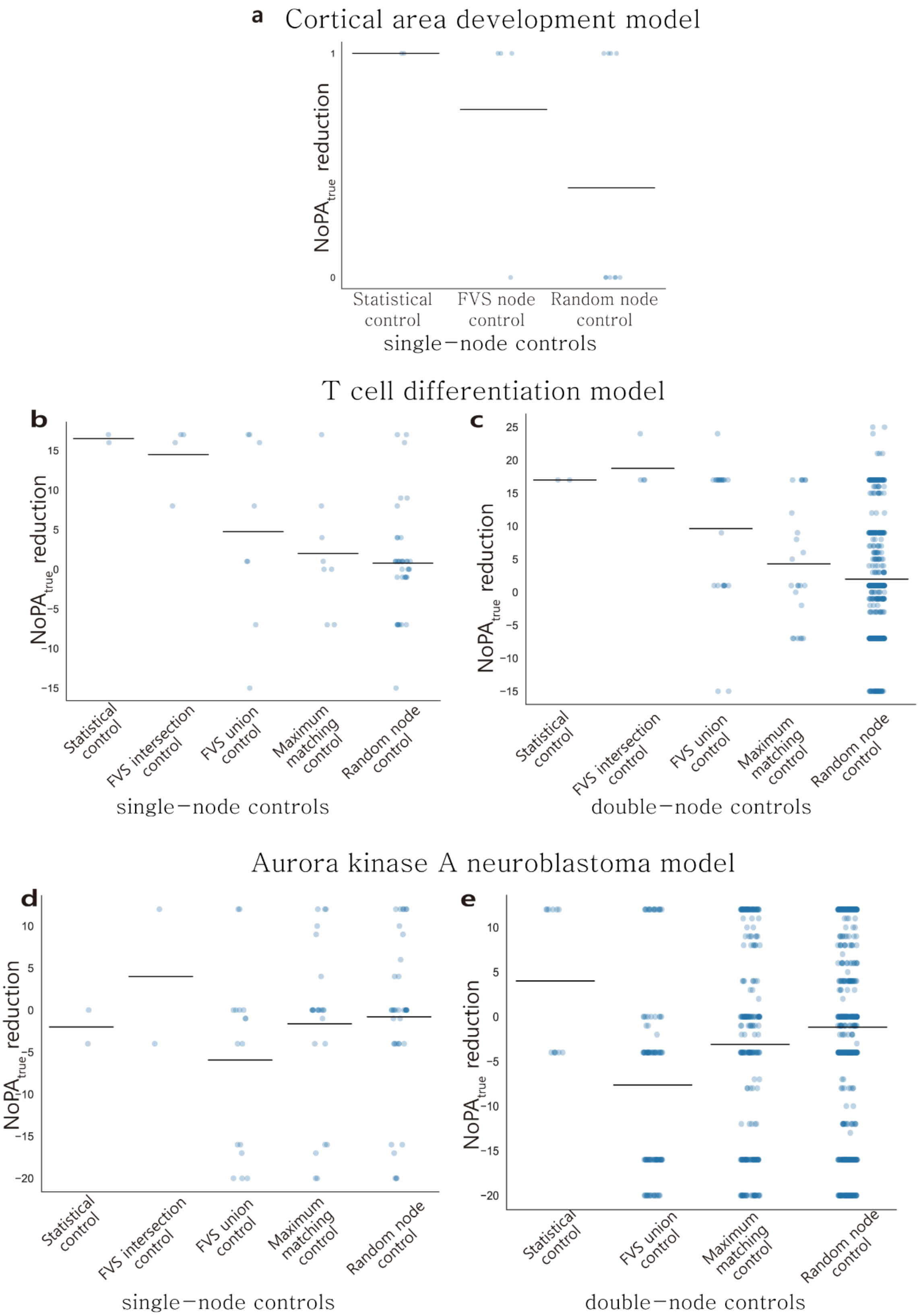
a
Cortical area development model
NoPA_true reduction
Statistical control
FVS node control
Random node control
single−node controls
T cell differentiation model
b
c
NoPA_true reduction
Statistical control
FVS intersection control
FVS union control
Maximum matching control
Random node control
single−node controls
double−node controls
Aurora kinase A neuroblastoma model
d
e
NoPA_true reduction
Statistical control
FVS intersection control
FVS union control
Maximum matching control
Random node control
single−node controls
double−node controls

Supplementary Figure S2. Comparison of top Statistical Control candidates with other structural control methods.

The statistical control (SC) result for control is compared to other control approaches across three biological networks. For single-node control (double-node control), one node (two node combination) is selected from the set of nodes and each node is randomly fixed to 0 or 1. Unlike Figures 3,4, and 5 in the main text, only the top candidate for SC is selected. Although this emphasizes an advantage of SC control, the average may be more susceptible to outliers due to small sample size. The intersection of FVS sets is also included despite its small sample size. $\mathrm{NoPA}_{\mathrm{true}}$ reduction is compared between control approaches. (**a**) In the cortical area development model, SC is superior to all other methods. (**b, c**) In the T cell differentiation model, for the single-node control the $\mathrm{NoPA}_{\mathrm{true}}$ reduction is higher than all other methods, and for double-node control it is only surpassed by FVS intersection control. (**d, e**) In the Aurora Kinase A neuroblastoma model, for the single-node control, the $\mathrm{NoPA}_{\mathrm{true}}$ reduction is highest for FVS intersection control, followed by SC control, whereas for double-node control SC produces a higher $\mathrm{NoPA}_{\mathrm{true}}$ reduction than any other approach.
